# Lipopolysaccharide-Induced Autophagy Mediates Retinal Pigment Epithelium Cells Survival. Modulation by the Phospholipase D Pathway

**DOI:** 10.3389/fncel.2019.00154

**Published:** 2019-04-24

**Authors:** Vicente Bermúdez, Paula Estefanía Tenconi, Norma María Giusto, Melina Valeria Mateos

**Affiliations:** ^1^Instituto de Investigaciones Bioquímicas de Bahía Blanca (INIBIBB), Consejo Nacional de Investigaciones Científicas y Técnicas (CONICET), Bahía Blanca, Argentina; ^2^Departamento de Biología, Bioquímica y Farmacia (DBByF), Universidad Nacional del Sur (UNS), Bahía Blanca, Argentina

**Keywords:** retinal pigment epithelium, lipopolysaccharide, autophagy, inflammation, phospholipase D

## Abstract

Inflammation and oxidative stress are common factors involved in the pathogenesis of retinal diseases, such as aged-related macular degeneration (AMD) and diabetic retinopathy (DR). Autophagy is a catabolic process essential to cell survival in response to stress. This process is highly active in retinal pigment epithelium (RPE) cells. Our previous findings demonstrated that lipopolysaccharide (LPS) induces an inflammatory response of RPE cells that implies classical phospholipases D (PLD1 and 2) activation, cyclooxygenase-2 (COX-2) expression, prostaglandin E_2_ (PGE_2_) production and reduced cell viability. In this work, we studied the autophagic process and its modulation by the PLD pathway in D407 and ARPE-19 RPE cells exposed to LPS. LPS (10 μg/ml or 25 μg/ml) exposure for 24 h increased light chain 3B-II (LC3B-II) content (an autophagy marker) and LC3B-positive punctate structures in both RPE cell lines studied. Next, the drug bafilomycin A_1_ (BAF, 50 nM) was used to block the autophagic flux. In cells pre-incubated with BAF, LC3B-II and sequestosome 1 (SQSTM1/p62) levels and autophagosome-like structures were increased by LPS, demonstrating that the inflammatory injury increases the autophagic process in RPE cells. To study the role of the PLD pathway, cells were pre-incubated for 1 h with selective PLD1 (VU0359595) or PLD2 (VU0285655-1) inhibitors prior to LPS addition. Under control condition, LC3B-positive punctate structures were increased in cells pre-incubated with PLD2 inhibitor while with PLD1 inhibitor were increased in cells exposed to LPS. MTT reduction assays showed that early autophagy inhibitors, 3-methyladenin (3-MA) or LY294002, enhanced the loss in cell viability induced by LPS exposure for 48 h. On the contrary, the inhibition of PLD1 and PLD2 prevented the loss in cell viability induced by LPS. In conclusion, our results show that even though LPS treatment promotes an inflammatory response in RPE cells, it also triggers the activation of the autophagic process which in turn may serve as a protective mechanism for the cells. In addition, we demonstrate that the PLD pathway modulates the autophagic process in RPE cells. Our findings contribute to the knowledge of the molecular basis of retinal inflammatory and degenerative diseases and open new avenues for potential therapeutic exploration.

## Introduction

The retinal pigment epithelium (RPE) is a monolayer of pigmented epithelial cells located between the retina and the vascular choroid, constituting the outer blood-retinal barrier (BRB). These epithelial cells play various critical roles for the correct function of the neural retina and photoreceptor (PR) survival, such as the secretion of several growth factors and cytokines and the transport of nutrients and water to the retina. Also, they protect against photo-oxidation and mediate the re-isomerization of all-trans-retinal and the renewal of photoreceptor outer segments (POS) by phagocytosis (Strauss, 2005; Strauß, 2016; Carr et al., 2009). Being a fundamental part in the BRB, RPE cells contribute to establishing the immunological privilege of the eye (Simó et al., 2010; Perez et al., 2013; Pavan et al., 2014). Likewise, RPE mediates in the immune response of the retina, by secreting pro- and anti-inflammatory cytokines, chemoattractants proteins, adhesion molecules and complement factors (Strauss, 2005; Strauß, 2016; Simó et al., 2010; Viringipurampeer et al., 2013).

Inflammation is a key factor in the pathogenesis of several retinal diseases such as age-related macular degeneration (AMD), diabetic retinopathy (DR), retinitis pigmentosa and uveitis, ultimately leading to varying degrees of vision loss (Shen et al., 2000; Rodrigues, 2007; Leung et al., 2009; Kauppinen et al., 2016). The inflammatory response is characterized by a series of reactions, including vasodilation and recruitment of immune cells and plasma proteins to the site of infection or tissue injury (Medzhitov, 2008; Ahmed, 2011; Liu et al., 2017). Inflammation can be both a protective and adaptive response while its deregulation can cause excessive or long-lasting tissue damages, contributing to the development of chronic inflammatory diseases (Medzhitov, 2008; Ahmed, 2011; Whitcup et al., 2013; Liu et al., 2017). It has been demonstrated that RPE cells can participate in the inflammatory response observed in several retinal degenerative diseases through the modification of its secretome (Arjamaa et al., 2017; Datta et al., 2017; Willermain et al., 2018). In view of the essential role of the RPE in PR viability and in visual function, elucidating the molecular mechanisms elicited by inflammation in this tissue could provide new insights for the treatment of retinal diseases.

Previous work from our laboratory demonstrated that the human RPE cell line ARPE-19 expresses both classical phospholipase D isoforms (PLD1 and PLD2) and that lipopolysaccharide (LPS) stimulates PLD activity (Mateos et al., 2014). Classical PLDs hydrolyze phosphatidylcholine (PC) to generate the lipid second messenger phosphatidic acid (PA) and choline (Exton, 2002; Foster and Xu, 2003; Peng and Frohman, 2012; Frohman, 2015). PA can be further dephosphorylated by lipid phosphate phosphatases (LPPs) in order to generate diacylglycerol (DAG), another lipid messenger (Brindley, 2004; Brindley et al., 2009). DAG and PA, as bioactive lipids, can modulate the activity of various proteins involved in cell signaling events, such as protein kinases C (PKC), protein kinase D (PKD), and the mammalian target of rapamycin (mTOR) complex, among others (Wang, 2006; Carrasco and Mérida, 2007; Newton, 2010; Foster et al., 2014). Through the modulation of these signaling proteins, DAG and PA participate in various cellular processes, such as vesicular trafficking, endo and exocytosis and cell survival.

Our previous study constituted the first evidence of classical PLDs participation in the LPS-induced inflammatory response of RPE cells through extracellular signal-regulated kinase (ERK1/2) activation and cyclooxygenase-2 (COX-2) expression (Mateos et al., 2014). Furthermore, we showed that PLD1 plays a dual role in LPS-exposed RPE cells, on the one hand by promoting cell damage through COX-2 induction and on the other by preventing LPS-induced apoptotic signals through PKCε modulation (Tenconi et al., 2016).

Autophagy is a conserved lysosomal self-digestion process ubiquitous in eukaryotic cells that respond to stress conditions, allowing them to adapt to environmental and developmental changes (Klionsky, 2007). Autophagy is initiated by the formation of double membrane-bound vesicles, called autophagosomes, which sequester cytoplasmic material. Finally, the fusion of autophagosomes with lysosomes allows the degradation of autophagic cargo and the subsequent recycling of nutrients and membranes (Yang and Klionsky, 2010). Autophagy is highly active in RPE and PR, being responsible for the lysosomal degradation and recycling of proteins and organelles (Wang et al., 2009). As a recycling process, autophagy has been described as a cellular pro-survival process, although other evidence indicates that under certain circumstances it may conduct to programmed cell death (Ferrington et al., 2016; Montagna et al., 2016; Deretic and Klionsky, 2018). Recently, evidences relating autophagy and inflammation have been described that may lead to therapeutic targeting (Netea-Maier et al., 2016), also in the RPE (Liu et al., 2016). Within the kinases that regulate the onset of autophagy, mTOR is the most important since the active mTORC1 complex prevents the formation of autophagosomes (Saxton and Sabatini, 2017).

Taking into account: (i) the participation of classical PLDs in the LPS-induced inflammatory process in RPE cells through ERK1/2 activation; (ii) the role the latter performs in mTOR regulation (Wang et al., 2017); and (iii) the role of PA and DAG signaling in autophagy (Dall’Armi et al., 2013), PLD possible participation in the regulation of the autophagic process in RPE cells exposed to an inflammatory context was further explored in the present work.

In view of the above, this study aims at arriving at a better understanding of the role of PA- and DAG-mediated signaling pathways in RPE cells exposed to inflammatory conditions.

## Materials and Methods

### Reagents

Triton X-100 (octyl phenoxy polyethoxyethanol), dimethyl sulfoxide (DMSO), LPS from *Klebsiella pneumoniae* (LPS, L4268), LY294002 (2-(4-Morpholinyl)-8-phenyl-1(4H)-benzopyran-4-one hydrochloride) and MTT (3-(4,5-dimethylthiazol-2-yl)-2, 5-diphenyltetrazolium bromide) were from Sigma-Aldrich (St. Louis, MO, USA). VU0359595 (PLD1i) and VU0285655-1 (PLD2i) were from Avanti Polar Lipids, Inc. (Alabaster, AL, USA). 4′,6-diamidino-2-phenylindole dihydrochloride (DAPI) was from Life Technologies Corporation (Grand Island, NY, USA). 3-methyladenine (3-MA), rapamycin (RAP) and bafilomycin A_1_ (BAF) were from Santa Cruz Biotechnology, Inc. (Santa Cruz, CA, USA). 5(6)-carboxy-2′7′-dichlorodihydrofluorescein diacetate (DCDCDHF), TO-PRO™-3 Iodide and DAPI were from Molecular Probes (Eugene, OR, USA). All other chemicals were of the highest purity available.

### Antibodies

Rabbit polyclonal antibody anti-light chain 3B (anti-LC3B; #2775) was from Cell Signaling (Beverly, MA, USA). Mouse monoclonal anti-SQSTM1/p62 (sc-28359) and rabbit polyclonal anti-nuclear factor kappa B (anti-NFκB) p65 (sc-109) antibody were purchased from Santa Cruz Biotechnology, Inc. (Santa Cruz, CA, USA). Mouse monoclonal anti-α Tubulin (DM1-A; CP06) was from EMD/Biosciences-Calbiochem (San Diego, CA, USA). Polyclonal horse radish peroxidase (HRP)-conjugated sheep anti-mouse IgG (NA931V) and polyclonal HRP-conjugated donkey anti-rabbit IgG (NA934V) were purchased from GE Healthcare (Malborough, MA, USA). Alexa Fluor^®488^ goat anti-rabbit (A11008) and Alexa Fluor^®488^ goat anti-mouse (A11001) were from Life Technologies Corporation (Grand Island, NY, USA).

### Retinal-Pigmented Epithelium Cell Cultures and Treatments

Two human retinal-pigmented epithelium cell lines (ARPE-19 and D407) were used in this work. ARPE-19 cells from the American Type Culture Collection (ATCC, Manassas, VA, USA) were generously donated by Dr. E. Politi and Dr. N. Rotstein (INIBIBB, Bahía Blanca, Argentina). D407 cells were a generous gift from Dr E. Rodriguez-Bouland (Weill Medical College of Cornell University, New York, NY, USA). ARPE-19 cells were maintained in Dulbecco’s Modified Eagle’s Medium (DMEM) supplemented with 10% fetal bovine serum (FBS, Natocor, Córdoba, Argentina) and antibiotic-antimycotic (Anti-Anti 100×, Gibco by Life Technologies) at 37°C under 5% CO_2_. D407 cells were maintained in 5% FBS DMEM. For western blot (WB) assays, cells were grown to 100% confluence on plastic 35 mm diameter culture dishes. Cell cultures were serum-starved for 30 min prior to LPS treatment with different concentrations (10 or 25 μg/ml) in serum-free DMEM or the same volume of sterile ultra pure water (control condition), for 24 or 48 h. LPS stock (4 mg/ml) was prepared in sterile ultra-pure water. Cells were pre-incubated with different concentrations (0.5 or 5 μM) of VU0359595 (PLD1i) to inhibit PLD1 activity or with different concentrations (0.5 or 5 μM) of VU0285655-1 (PLD2i) to inhibit PLD2 activity for 30 min at 37°C prior to cell stimulation with LPS. To inhibit the autophagic process cells were pre-incubated with 3-MA (5 mM) or with LY294002 (10 μM) for 30 min prior to LPS treatment. To block autophagosome fusion with lysosomes cells were pre-incubated with BAF (50 nM) for 30 min at 37°C prior to LPS treatment. As a positive control, cells were treated with the mammalian target of rapamycin complex 1 (mTORC1) inhibitor rapamycin (RAP, 1 μM) for 24 h in order to induce autophagy. DMSO (vehicle of the inhibitors) was added to all conditions to achieve a final concentration of 0.05%.

### MTT Reduction Assay

Cell viability was measured in terms of mitochondrial function. D407 cells (1.5 × 10^4^ cells/well) were seeded in 96-well plates. Cells were pre-incubated with different inhibitors as described above and after a 48 h LPS treatment, mitochondrial function was assessed by MTT reduction assay. MTT is reduced by mitochondrial dehydrogenases of metabolically viable cells to a colored, water-insoluble formazan salt. MTT (5 mg/ml) was prepared in phosphate buffer saline (PBS) and was added to the cell culture medium at a final concentration of 0.5 mg/ml. The culture plates were incubated for 1 h at 37°C in a 5% CO_2_ atmosphere, cells were then washed twice with PBS and lysed with 100 μl of a buffer containing 10% Triton X-100 and 0.1N HCl in isopropanol. The extent of MTT reduction was measured spectrophotometrically (570 nm absorbance–650 nm absorbance) using a Multiskan™ 60 microplate spectrophotometer (Thermo Fisher Scientific, Waltham, MA, USA). Results are expressed as arbitrary units (AUs) with respect to the control condition.

### Western Blot (WB)

WB assays were performed as previously described (Mateos et al., 2014). Briefly, after experimental treatment the medium was removed from confluent 35 mm dishes, cells were washed with PBS and scraped off with 80 μl ice-cold RIPA lysis buffer [10 mM Tris-HCl (pH 7.4), 15 mM NaCl, 1% Triton X-100, 5 mM NaF, 1 mM Na_2_VO_4_ and the complete protease inhibitor cocktail]. Protein content of total cell lysates was determined by the Bradford method (Bradford, 1976; Bio-Rad Life Science group, Hercules, CA, USA, #500-0006) and samples were denatured with Laemmli sample buffer at 100°C for 5 min (Laemmli, 1970). Thirty micrograms protein were separated by sodium dodecylsulfate polyacrylamide gel electrophoresis (SDS-PAGE) on 10% or 16% polyacrylamide gels and transferred to polyvinylidene fluoride (PVDF) membranes (Millipore, Bedford, MA, USA). Membranes were blocked with 10% bovine serum albumin (BSA) in TTBS buffer [20 mM Tris–HCl (pH 7.4), 100 mM NaCl and 0.1% (w/v) Tween 20] at room temperature for 2 h and subsequently incubated with primary antibodies overnight at 4°C. After three washes with TTBS, membranes were exposed to the appropriate HRP-conjugated secondary antibody for 2 h at room temperature. Immunoreactive bands were detected by enhanced chemiluminescence (Pierce^®^ECL Western Blotting Substrate, #32209, Thermo scientific) using UltraCruz# Autoradiography Film, Santa Cruz Biotechnology, Inc. (Santa Cruz, CA, USA). Densitometry values of the immunoreactive bands were determined using ImageJ 1.46 software (NIH). The molecular weight of bands was determined using the spectra multicolor broad range protein ladder (26634, Thermo Scientific).

### Measurement of Reactive Oxygen Species (ROS) Production

Reactive oxygen species (ROS) production was measured using the probe DCDCDHF (Molecular Probes, Eugene, OR, USA). This probe can cross the membrane and, after oxidation, it is converted into a fluorescent compound. 6 × 10^4^ D407 cells were seeded onto 12 mm coverslips and exposed to LPS (10 μg/ml) or to control condition for 24 h. After the experimental treatment, the cell culture medium was removed and replaced by medium containing 10 μM DCDCDHF and cells were incubated for 30 min at 37°C. Cells were subsequently washed three times with PBS and coverslips were mounted for examination with a Nikon Eclipse TE2000-S microscope coupled to a Nikon DS-Qi2 camera (1,608 × 1,608 pixels) and a 60× Plan Apo (1.4 N.A.) oil-immersion objective. Fluorescence intensity values were determined using ImageJ 1.46 software.

### Immunocytochemistry and Fluorescence Microscopy

For immunocytochemistry assays, 6 × 10^4^ cells were seeded onto 12 mm coverslips on 24-well plates. After LPS treatment (10 or 25 μg/ml) for 24 h cells were washed twice with ice-cold PBS and fixed with methanol for 15 min at −20°C. Cells were then blocked with 2% BSA in PBS for 15 min and exposed to primary anti-LC3B antibody (1:200 in blocking solution), anti-p62 (1:100 in blocking solution) or anti-NFκB p65 (1:100 in blocking solution). Cells were then exposed to the appropriate Alexa Fluor^®488^-conjugated secondary antibodies (1:500 in blocking solution). Finally, nuclei were stained with DAPI or TO-PRO-3 for 10 min at room temperature. The whole immunocytochemical method was performed at room temperature and three washes with ice-cold PBS were performed between each step of the procedure and after nuclear staining. Coverslips were mounted for examination with a Nikon Eclipse TE2000-S microscope coupled to a Nikon DS-Qi2 camera (1,608 × 1,608 pixels) and a 60× Plan Apo (1.4 N.A.) oil-immersion objective or with a TCS-SP2 confocal microscope (Leica Mikrosysteme Vertrieb GmbH, Wetzlar, Germany) equipped with an acousto optical beam splitter using a 63× (1.2 N.A.) objective. Fluorescence intensity values were determined using ImageJ 1.46 software.

### Data Quantification and Statistical Analysis

In immunocytochemistry images, the percentage of cells with LC3B- or p62- positive punctate structures was manually scored by a blinded operator. With this aim, at least 100 cells of each condition were counted. Data shown in bar graphs represent the percentage mean value ± SD of four independent experiments (*n* = 4). Fluorescence intensity values from immunocytochemistry images and densitometry values of the WB immunoreactive bands were determined using ImageJ 1.46 software. WBs shown are representative images of samples from four independent experiments (*n* = 4).

Statistical analysis of the data was performed using ANOVA followed by Tuckey’s test to compare means or Student’s *t*-test when only two conditions were compared. *p*-values lower than 0.05 were considered statistically significant.

## Results

### LPS Induces NFκB Nuclear Translocation, ROS Generation and Reduces Cell Viability in D407 RPE Cells

Our previous results demonstrated that the exposure of ARPE-19 cells to LPS induces an inflammatory response that engages NO production, PLD activation, COX-2 expression, prostaglandin E2 (PGE_2_) secretion and reduced cell viability (Mateos et al., 2014). To further characterize the inflammatory response of RPE cells induced by LPS, immunocytochemistry assays were performed in order to evaluate NFκB translocation to the nucleus. In addition, ROS generation was evaluated by using the probe DCDCDHF.

**Figure 1:**
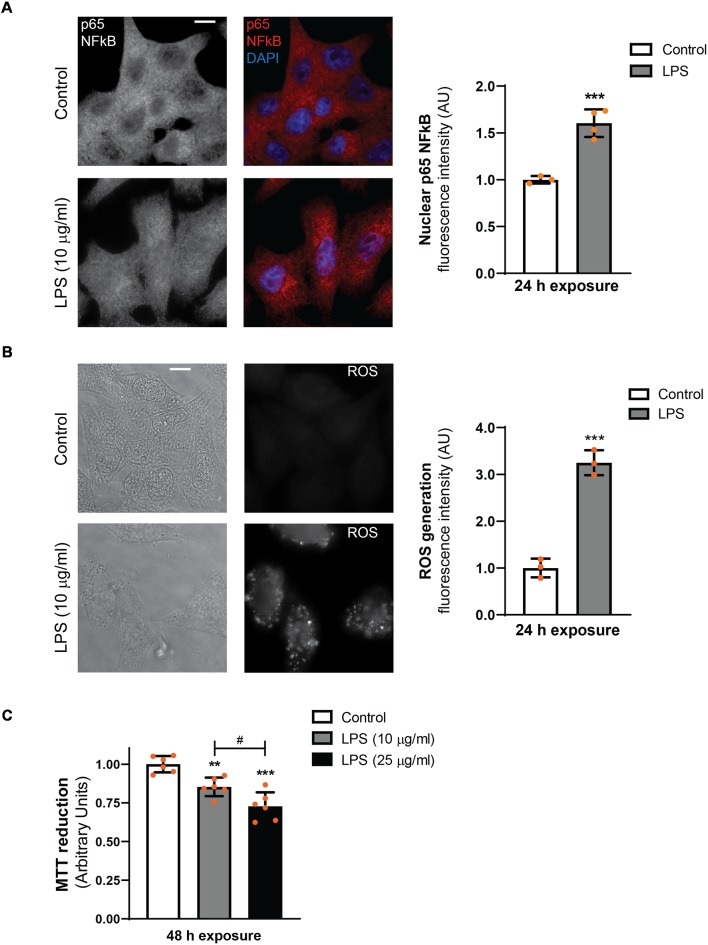
Lipopolysaccharide (LPS) inflammatory model in D407 cells. **(A)** Immunofluorescence assays for analysis of nuclear factor kappa B (NFκB; p65) subcellular distribution were performed as described in “Materials and Methods” section in D407 cells exposed to LPS (10 μg/ml) or to control condition (ultrapure water) for 24 h. Results are expressed as arbitrary units (AU). **(B)** Reactive oxygen species (ROS) generation was analyzed in D407 cells treated with LPS (10 μg/ml) or under control condition (ultrapure water) for 24 h as detailed in “Materials and Methods” section. Left panel show light microscopy images of the cells. For **(A,B)**, bar graph shows fluorescence intensity expressed as arbitrary units with respect to control conditions (mean ± SD). Dots indicate individual values of different experiments (*n* ≥ 3). Asterisks (*) indicate significant differences with respect to control condition (***p < 0.0001). Scale bar = 10 μm. **(C)** Cell viability was evaluated using the MTT reduction assay in D407 cells treated with LPS (10 or 25 μg/ml) or ultrapure water (control condition) for 48 h. Results are expressed as arbitrary units. Dots indicate individual values from six different experiments (*n* = 6). Asterisks (*) indicate significant differences with respect to control condition (**p < 0.001; ***p < 0.0001). Number signs (#) indicate significant differences between LPS conditions (^#^*p* < 0.05).

In D407 cells exposed to LPS (10 μg/ml) for 24 h epifluorescence microscope images show an increment in NFκB (p65) nuclear translocation (by 60%) and ROS generation (by 220%) with respect to control condition (Figures 1A,B). Similar results were obtained when p65 nuclear translocation was observed by confocal microscopy (Supplementary Figure S1). Our previous findings in ARPE-19 cells showed that cell viability was affected only after a 48 h exposure to LPS (Mateos et al., 2014). Similar results were obtained in D407 RPE cells, in which cell viability was reduced by 15% and by 27% in cells exposed to 10 and 25 μg/ml for 48 h, respectively (Figure 1C). These results together with our previous findings demonstrate that LPS treatment induces a typical inflammatory response of RPE cells. Taking all these data into account, we next used both RPE cell lines (D407 and ARPE-19) to study the role of the autophagic process in the LPS-induced RPE inflammatory response.

### LPS Exposure Induces an Increment in LC3-II Content in RPE Cells

**Figure 2:**
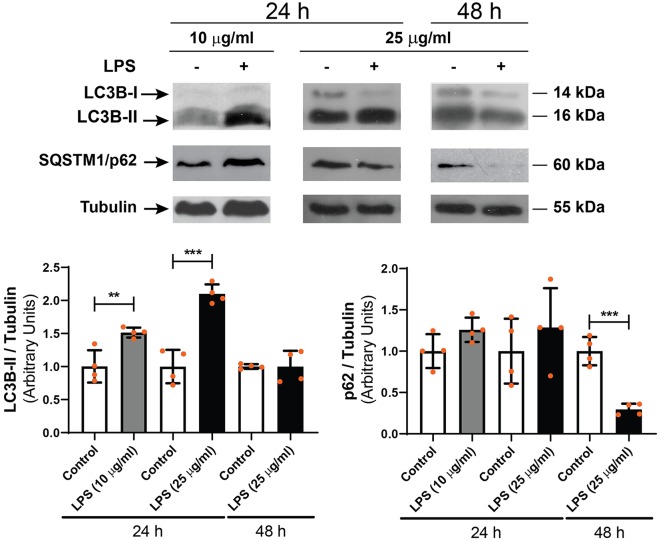
Western blot (WB) assays were performed in order to determine light chain 3B-II (LC3B-II) and p62 levels in D407 cells exposed to LPS (10 or 25 μg/ml) or to control condition (ultrapure water), for 24 or 48 h. Numbers to the right indicate molecular weights designated by standard markers. Bar graphs show the densitometry values of LC3B-II/α-Tubulin or p62/α-Tubulin. Results are expressed as arbitrary units. Dots indicate individual values from four independent experiments (*n* = 4). Asterisks (*) indicate significant differences with respect to each control condition (**p < 0.01; ***p < 0.0001).

To study the effect of LPS on the autophagic process of RPE cells, D407 cells were exposed to LPS (10 or 25 μg/ml) for 24 or 48 h and WB assays were performed in order to determine microtubule-associated protein 1 light chain 3B-II (LC3B-II) and sequestosome 1 (SQSTM1/p62) levels. WB showed that 10 and 25 μg/ml LPS increased LC3B-II content by 51% and by 100% with respect to control condition after 24 h treatment, respectively (Figure 2). No changes in LC3B-II content were detected after a 48 h exposure to LPS (25 μg/ml; Figure 2). SQSTM1/p62 content was not significantly changed after 24 h treatment with both LPS concentrations but was reduced by 70% after a 48 h LPS exposure (Figure 2).

**Figure 3:**
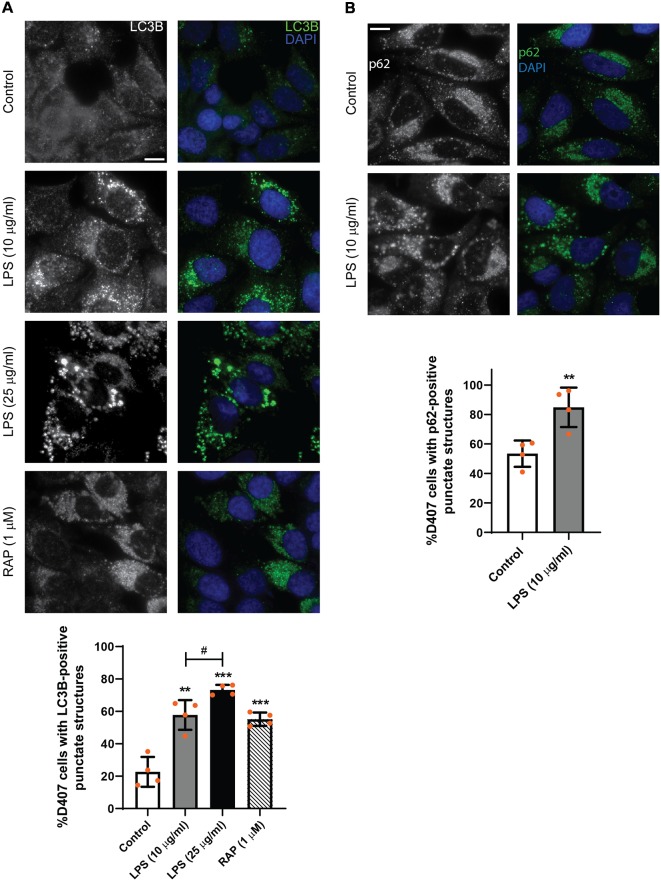
Immunofluorescence assays of D407 cells were performed as described in “Materials and Methods” section. **(A)** LC3B-positive punctate structures were analyzed in D407 cells exposed to LPS (10 or 25 μg/ml) or to control condition (vehicle). The mTORC1 inhibitor rapamycin (RAP, 1 μM) was used as positive control. Bar graph shows the percentage of cells presenting LC3B-positive punctate structures in each condition. Cells showing punctate structures similar or bigger to those observed in RAP condition were considered positives. Graph shows the mean percentage ± SD. Dots indicate individual values from four independent experiments (*n* = 4). **(B)** p62-positive punctate structures were analyzed in D407 cells exposed to LPS (10 μg/ml) or to control condition (ultrapure water) for 24 h. Bar graph shows the % of cells presenting p62-positive punctate structures in each condition. Graph shows the mean percentage ± SD. Dots indicate individual values from four independent experiments (*n* = 4). For **(A,B)**, cells were stained with DAPI to visualize the nuclear structure. Scale bar = 10 μm. Asterisks (*) indicate significant differences with respect to control condition (**p < 0.01; ***p < 0.001). Number signs (#) indicate significant differences between LPS conditions (^#^*p* < 0.05).

In order to study the subcellular distribution of autophagy markers LC3B and p62, immunocytochemistry assays were performed as described in “Materials and Methods” section. Figure 3 shows that LPS (10 or 25 μg/ml) treatment for 24 h increased LC3B-positive punctate structures in D407 cells (Figure 3A) and similar results were observed in ARPE-19 cells exposed to 25 μg/ml LPS for 24 h (Supplementary Figure S2). Under control condition, 22% of D407 cells presented LC3B-positive punctate structures and this percentage raised to 58% in cells treated with 10 μg/ml LPS and to 73% in cells treated with 25 μg/ml LPS (Figure 3A). The mTORC1 inhibitor rapamycin (RAP, 1 μM) was used as positive control (Figure 3A) and no significant differences were detected in LC3B-punctate structure formation between cells incubated with or without FBS (data not shown). Furthermore, larger LC3B-punctate structures were observed in D407 cells exposed to 25 μg/ml LPS with respect to cells exposed to 10 μg/ml LPS (Figure 3A). Although WB assays showed no significant changes in p62 content after a 24 h exposure to LPS (Figure 2), immunocytochemistry assays showed that a 24 h LPS (10 μg/ml) treatment increases significantly the percentage of D407 cells with p62-positive punctate structures (Figure 3B). These results demonstrate that LPS enhances autophagosome-like structures formation in RPE cells.

In view of our results, most of the following experiments were performed with 10 μg/ml LPS since this concentration induced an inflammatory-like response in RPE cells and increased LC3B-II content and autophagosome-like structures formation with fewer effects on cell metabolic state and viability.

**Figure 4:**
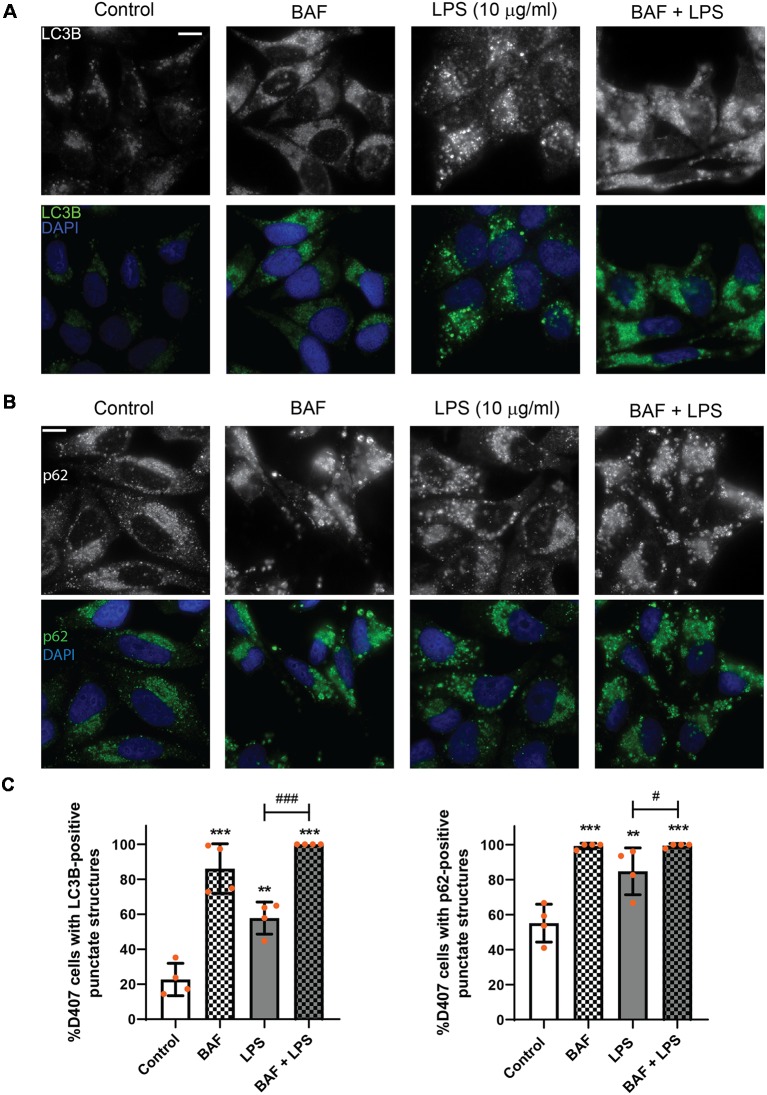
Evaluation of autophagic flux in LPS treated retinal pigment epithelium (RPE) cells. RPE cells were pre-incubated with bafilomycin A_1_ (BAF, 50 nM) or with vehicle dimethyl sulfoxide (DMSO) prior to incubation with LPS (10 μg/ml) or under control condition for 24 h. **(A,B)** Immunofluorescence assays were performed in D407 cells as described in “Materials and Methods” section to determine LC3B-positive **(A)** and p62-positive **(B)** punctate structures. Cells were stained with 4′,6-diamidino-2-phenylindole (DAPI) to visualize the nuclear structure. Scale bar = 10 μm. **(C)** Bar graphs show the percentage of cells presenting LC3B- or p62-positive punctate structures in each condition. Graphs show the mean percentage ± SD. Dots indicate individual values from four independent experiments (*n* = 4). Asterisks (*) indicate significant differences with respect to control condition (**p < 0.01; ***p < 0.001). Number signs (#) indicate significant differences between indicated conditions (^#^*p* < 0.05; ^###^*p* < 0.001).

**Figure 5:**
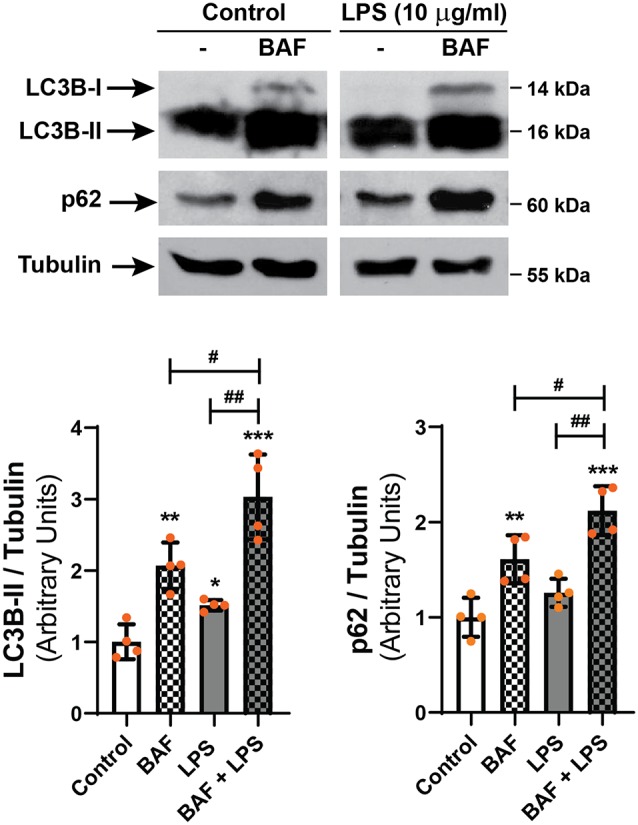
D407 cells were pre-incubated with BAF (50 nM) or with vehicle (DMSO) prior to incubation with LPS (10 μg/ml) or under control condition for 24 h. WBs were performed and analyzed as described in “Materials and Methods” section in order to determine LC3B-II and p62 levels. Numbers to the right indicate molecular weights designated by standard markers. Bar graphs show the densitometry values of LC3B-II/α-Tubulin or p62/α-Tubulin. Dots indicate individual values from four independent experiments (*n* = 4). Results are expressed as arbitrary units. Asterisks (*) indicate significant differences with respect to each control condition (*p < 0.05; **p < 0.01; ***p < 0.001). Number signs (#) indicate significant differences between indicated conditions (^#^*p* < 0.05; ^##^*p* < 0.01).

To discern whether LPS treatment induces the autophagic process or blocks the autophagic flux, D407 cells were pre-incubated with BAF (50 nM) in order to block autophagosome fusion with lysosomes and thus, autolysosome formation, prior to LPS (10 μg/ml) treatment for 24 h. In D407 cells, the blockage of the autophagic flux with BAF increased to 86 the percentage of cells with LC3B-positive punctate structures (Figures 4A,C) and to 98 the percentage of cells with p62-positive punctate structures under control condition (Figures 4B,C). 100% of the cells pre-incubated with BAF and exposed to LPS for 24 h presented LC3B-positive punctae and p62-positive punctae (Figures 4A–C). In addition, WBs showed that pre-incubation with BAF induces an increment in LC3B-II and p62 content under control condition and this increment was higher in cells pre-incubated with BAF and exposed to LPS for 24 h (Figure 5). Same results were observed in ARPE-19 cells (Data not shown). These results demonstrate that LPS treatment is not blocking the autophagic flux but is inducing the autophagic process in RPE cells.

### PLD1 and PLD2 Inhibition Modulates the Autophagic Process of RPE Cells

**Figure 6:**
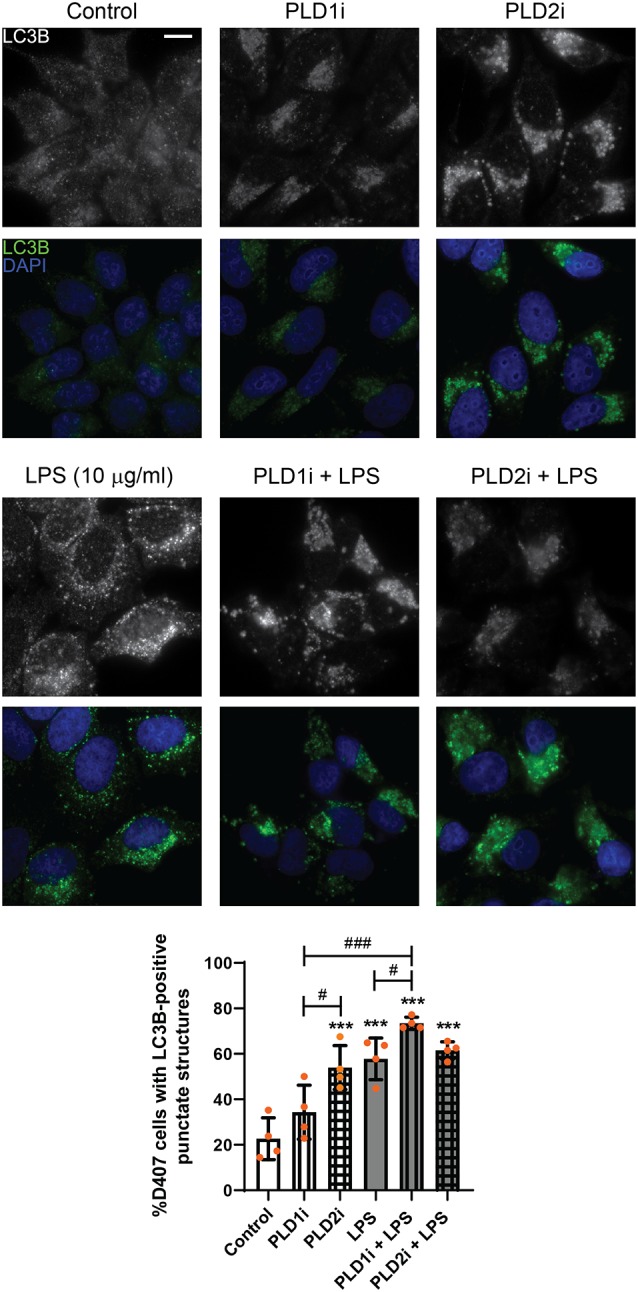
Role of phospholipase D (PLD) isoforms 1 and 2 in the autophagic process of RPE cells. D407 cells were pre-incubated with PLD1 or PLD2 selective inhibitors (PLD1i and PLD2i, 5 μM) or vehicle (DMSO) prior to incubation with LPS (10 μg/ml) or under control condition for 24 h. Immunofluorescence assays were performed to determine LC3B-positive punctate structures. Cells were stained with DAPI to visualize the nuclear structure. Scale bar = 10 μm. Bar graph shows the percentage of cells presenting LC3B-positive punctate structures in each condition. Graph shows the mean percentage ± SD. Dots indicate individual values from four independent experiments (*n* = 4). Asterisks (*) indicate significant differences with respect to control condition (***p < 0.001). Number signs (#) indicate significant differences between indicated conditions (^#^*p* < 0.05; ^###^*p* < 0.001).

**Figure 7:**
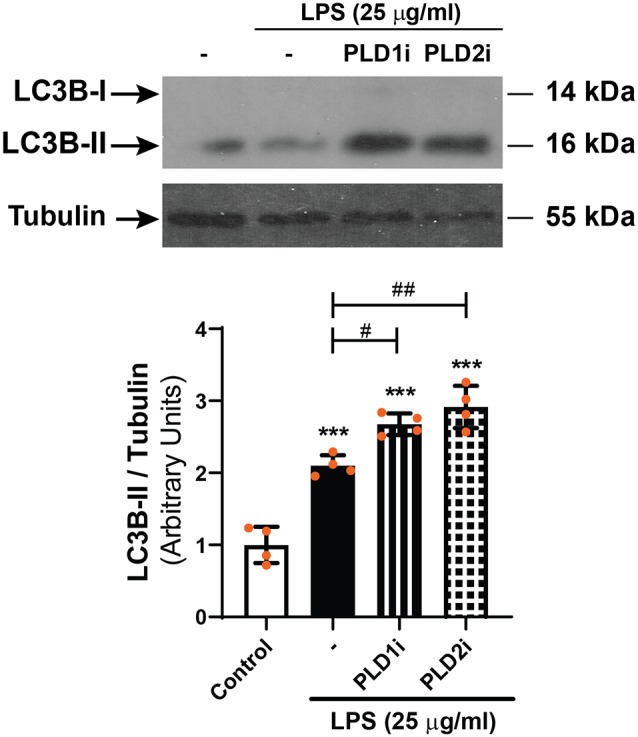
WBs were performed and analyzed as described in “Materials and Methods” section in order to determine LC3B-II levels in D407 cells pre-incubated with PLD1i (0.5 μM), PLD2i (0.5 μM) or vehicle (DMSO) prior to incubation with LPS (25 μg/ml)or under control condition for 24 h. The bar graph shows the densitometry values of LC3B-II/α-Tubulin. Dots indicate individual values from four independent experiments (*n* = 4). Results are expressed as arbitrary units. Asterisks (*) indicate significant differences with respect to control condition (***p < 0.001). Number signs (#) indicate significant differences with respect to LPS control condition (^#^*p* < 0.05; ^##^*p* < 0.01).

As stated above, our previous findings demonstrated the participation of classical PLDs in the LPS-induced inflammatory response of RPE cells (Mateos et al., 2014). To study the role of PLD1 and PLD2 in the LPS-induced autophagic process, D407 cells were pre-incubated with PLD1 or PLD2 selective inhibitors (PLD1i and PLD2i, 5 μM) prior to LPS treatment for 24 h, as described in “Materials and Methods” section. Immunofluorescence assays showed that under control condition the percentage of D407 cells with LC3B-positive punctate structures was increased significantly (to 53%) in cells pre-incubated with PLD2i while PLD1i increased this percentage (to 73%) only in cells exposed to LPS (Figure 6). To further support these results, WBs assays show that PLD1 and PLD2 inhibition (with lower inhibitor concentrations than those used for immunofluorescence assays, 0,5 μM) also increased LC3B-II content in D407 cells exposed to a higher LPS concentration (25 μg/ml) for 24 h (Figure 7). These results suggest that PLD2 modulates basal autophagy under control condition while both PLDs modulate the autophagic process under LPS-induced inflammatory conditions.

### The Activation of the Autophagic Process Protects RPE Cells From the Inflammatory Injury

**Figure 8:**
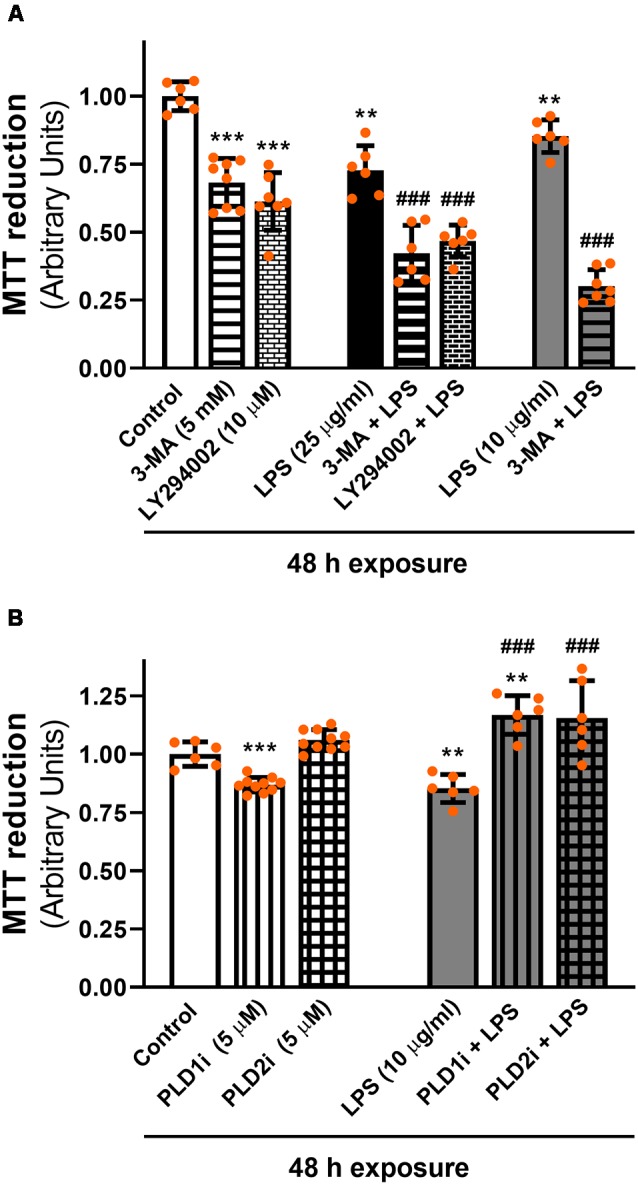
**(A)** Effect of autophagy inhibitors on cell viability. D407 cells were pre-incubated with autophagy inhibitor 3-methyladenine (3-MA), LY294002 or vehicle (DMSO) prior to LPS (10 or 25 μg/ml) or control condition exposure for 48 h. **(B)** Effect of PLD inhibitors on cell viability. D407 cells were pre-incubated with PLD1i (5 μM), PLD2i (5 μM) or vehicle (DMSO) prior to LPS (10 μg/ml) or control condition exposure for 48 h. For **(A,B)**, cell viability was evaluated using the MTT reduction assay, as described in “Materials and Methods” section expressed as arbitrary units. Dots indicate individual values from different experiments (*n* ≥ 6). Asterisks (*) indicate significant differences with respect to control condition (vehicle; **p < 0.01; ***p < 0.0001). Number signs (#) indicate significant differences with respect to each LPS (vehicle) condition (^###^*p* < 0.0001).

To assess the role of the autophagic process in LPS-induced cell damage, D407 cells were pre-incubated with autophagy inhibitors 3-MA and LY294002 prior to LPS exposure for 48 h since, as stated above, we previously demonstrated that RPE cell viability is not affected after shorter exposures to LPS (Mateos et al., 2014). These inhibitors block autophagy at an early stage by inhibiting class III phosphatidylinositol 3-kinase (PI3K; Klionsky et al., 2016). Figure 8A shows that under control condition 3-MA (5 mM) and LY294002 (10 μM) reduced D407 cell viability by 32% and by 39%, respectively. Furthermore, both inhibitors enhanced the cell viability loss induced by LPS since MTT reduction was reduced by 58% and by 54% in cells pre-incubated with 3-MA and LY294002 and exposed to LPS (25 μg/ml) for 48 h, respectively. Cell viability loss was also enhanced by 70% in cells pre-incubated with 3-MA and exposed to a lower LPS concentration (10 μg/ml) for 48 h (Figure 8A). On the contrary, the inhibition of PLD1 and PLD2 with 5 μM PLD1i or PLD2i prevented the loss in cell viability induced by LPS (10 μg/ml) in D407 cells (Figure 8B). Lower concentrations (0.5 μM) of PLD1i and PLD2i did not prevent the LPS-induced viability loss of D407 cells (Data not shown). Our results show that the autophagic process is a protective mechanism triggered under LPS-induced inflammation in RPE cells.

## Discussion

LPS can reach and directly stimulate RPE cells in situations of acute ocular inflammation, such as bacterial endophthalmitis (posterior segment eye infection) and uveitis (Leung et al., 2009; Pollreisz et al., 2012). Endophthalmitis can arise after an eye surgery, intravitreal injections, trauma and sepsis (endogenous endophthalmitis), the latter mostly seen in immunocompromised patients (Pollreisz et al., 2012; Haddock et al., 2014; Holland et al., 2014). Although bacterial endophthalmitis is an unusual pathology, it is a vital ocular emergency since it usually ends up in vision loss (Haddock et al., 2014; Holland et al., 2014). Additionally, LPS is often used to induce an inflammatory response in cell cultures. In line with this, our previous findings demonstrated that the exposure of ARPE-19 cells to LPS induces a typical inflammatory response which involves PLD and ERK1/2 activation, COX-2 expression, PGE_2_ secretion, reduced cell viability and increased caspase-3 cleavage (Mateos et al., 2014; Tenconi et al., 2016). In the present work, we demonstrate that the LPS-induced inflammatory response of RPE cells also involves NFκB nuclear translocation and ROS generation.

Autophagy is a very active catabolic process in RPE cells. It is essential for cell survival in response to stress (Frost et al., 2014; Sinha et al., 2016). Different studies reported that autophagy is selectively dysregulated in the RPE from AMD patients and that downregulation of the autophagic pathway renders the RPE more susceptible to oxidative stress while increased autophagic flux protected the RPE from oxidative damage (Mitter et al., 2014; Golestaneh et al., 2017). In this work, we demonstrate that LPS increases autophagy in RPE cells mediating cell survival. Furthermore, our results show that the PLD pathway can modulate the autophagic process.

Once autophagy is initiated the cytosolic form of LC3 (LC3-I) is processed and transformed by the addition of a group of phosphatidylethanolamine to form LC3-II. This lipidation reaction permits the recruitment of the protein to autophagosome membranes. There are three human isoforms of LC3, LC3A, LC3B, and LC3C. Among the three isoforms, LC3B has been the most studied and has become one of the most reliable markers for characterization of the autophagic process (Klionsky et al., 2016). Similarly, SQSTM1/p62 by interaction with LC3 is associated with the autophagosomal membrane to facilitate the degradation of ubiquitinated protein aggregates (Rubinsztein et al., 2009; Ferrington et al., 2016; Rosa et al., 2016). Finally, p62 is degraded together with ubiquitinated protein in the autolysosome (Rubinsztein et al., 2009; Ferrington et al., 2016; Rosa et al., 2016). Thus, an increase in LC3B-II content and decreased p62 levels are associated with autophagic activation, although p62 changes can be cell type, time and context specific (Klionsky et al., 2016).

The increase in LC3B-II content and in the percentage of cells with LC3B- and p62-positive punctate structures demonstrates that a 24 h LPS exposure induces autophagy in RPE cells. Furthermore, the reduced p62 content observed after a 48 h LPS exposure and the results obtained in the presence of BAF confirm that LPS treatment is inducing the autophagic process in RPE cells rather than blocking its flux. In agreement with our results, Wang and collaborators reported that in ARPE-19 cells LPS plus tetrachlorodibenzo-*p*-dioxin induces the formation of autophagosome-like structures observed by transmission electron microscopy (Wang et al., 2016). The inhibition of autophagy with 3-MA and LY294002, which target early events in the autophagy cycle through irreversible inhibition of class III PI3K, worsened LPS-induced viability loss demonstrating that autophagy is a protective mechanism elicited under LPS condition in RPE cells. Moreover, early autophagy inhibition reduced RPE cell viability in our control condition suggesting that autophagy in a basal context is important for RPE cell maintenance.

The PLD pathway has been postulated both as a positive as well as a negative autophagy modulator (Gomez-Cambronero and Kantonen, 2014). It has been demonstrated that PLD-generated PA induces membrane curvatures necessary for phagocytosis as well as for the formation of the initial autophagosome (Dall’Armi et al., 2010; Holland et al., 2016). On the contrary, PLD-generated PA activates mTORC1, the main inhibitor of the autophagy initiation machinery (Sun and Chen, 2008; Foster et al., 2014; Munson and Ganley, 2015). Moreover, the PLD-mediated autophagic regulation has been proposed as a potential target for cancer therapy (Jang et al., 2014). PA was also shown to bind and activate the mTOR effector S6 kinase (S6K) independently of mTOR (Lehman et al., 2007). An interrelation between PLD2 and mTOR/S6K has been demonstrated in HL-6 leukemia cells, where PLD2 was shown to mediate IL-8-induced mTOR/S6K mRNA up-regulation and, in reverse direction, mTOR and S6K were shown to down-regulate PLD2 activity and gene expression (Tabatabaian et al., 2010). Furthermore, inhibition and genetic knockdown of PLD2 significantly induced autophagy in HT29 and HCT116 colorectal cancer cells (Hwang et al., 2014). However, the role of the PLD pathway in RPE cells autophagic process was a complete unstudied research field. The novelty of our findings is that we show that under control (basal) condition autophagosome-like structures were increased in cells pre-incubated with PLD2i while PLD1i increased autophagosome-like structures in cells exposed to LPS. Our results constitute the first evidence that the PLD pathway is a negative autophagy modulator in RPE cells, possibly through PLD-mediated PA generation and mTORC1 activation. Whereas PLD2 modulates basal autophagy under control condition, both PLDs inhibit the autophagic process under LPS conditions. The PLD2-modulation of basal autophagy in RPE cells is in agreement with the fact that, of both classical PLD isoforms, PLD2 presents higher basal activity.

**Figure 9:**
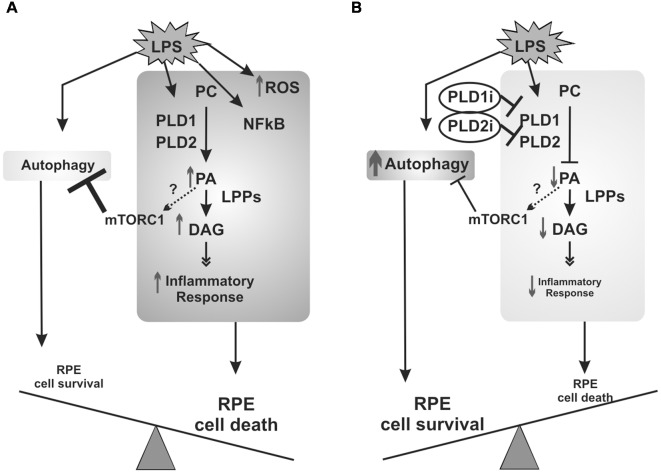
Schematic view of signaling events elicited by LPS in RPE cells. **(A)** PLD activation, ROS generation and NFκB nuclear translocation are part of the inflammatory signaling cascade induced in RPE cells exposed to LPS that ultimately leads to cell viability loss. RPE cells answer to LPS also involves the induction of the autophagic process which mediates cells survival. **(B)** When PLD1 and PLD2 are inhibited, the inflammatory response of RPE cells is reduced and autophagy is enhanced, possibly through a reduced phosphatidic acid (PA)-dependent mTORC1 activation, leading to RPE cell survival. Dashed arrows and question marks indicate possible but unstudied mechanisms.

In agreement with results obtained in RPE cells incubated with early autophagy inhibitors (which worsen cell viability loss), PLD inhibitors (which increase autophagy) prevented LPS-induced viability loss in D407 RPE cells. Together, our findings demonstrate that autophagy is a protective mechanism elicited under the LPS-induced inflammatory response of RPE cells. Certainly, the protective effect of PLD inhibitors does not reside only in the modulation of autophagy since our previous findings demonstrated that the pharmacological inhibition of classical PLDs prevents LPS-induced COX-2 expression and PGE_2_ production in ARPE-19 cells (Mateos et al., 2014). To sum up, our previous work and the findings reported herein demonstrate that in RPE cells LPS induces an inflammatory-like response that involves PLD activation, ROS generation and NFκB nuclear translocation, leading to cell viability loss. Furthermore, LPS induces RPE autophagic process which mediates cells survival (Figure 9A). The inhibition of the PLD pathway not only reduces the inflammatory response but also enhances autophagy in RPE cells, possibly through a reduced PA-dependent mTORC1 activation, leading to RPE cell survival (Figure 9B).

It is well-recognized the role of NFκB in the regulation of inflammatory responses, by mediating the induction of various pro-inflammatory genes, the activation and differentiation of inflammatory T cells and the activation of inflammasomes (Liu et al., 2017). Furthermore, several studies have linked NFκB signaling with autophagy. It has been reported that NFκB is involved in advanced glycation end-products (AGE)-induced autophagy in diverse cell lines (Verma and Manna, 2016, 2017) and in the autophagic process observed in lung tissues from rats with induced pulmonary arterial hypertension (Zhai et al., 2018). Moreover, in several cell lines NFκB (p65) was shown to induce the expression of beclin1 (BECN1), a central protein that assembles the class III PI3K complex to trigger macroautophagy (Copetti et al., 2009; Han et al., 2018). In LPS-primed macrophages, NFκB was shown to upregulate p62 expression (Zhong et al., 2016) and in head and neck squamous cell carcinoma cells the expression of BECN1 and LC3 was also demonstrated to be modulated by NFκB (p65; He et al., 2017). However, the link between NFκB activation and the expression of autophagy-related proteins in the RPE has not been studied yet. Our results demonstrate that in RPE cells NFκB nuclear translocation and autophagy flux are triggered by LPS at the same time frame, opening interesting questions about the role of this transcription factor in RPE autophagic process.

In conclusion, our results showed that LPS treatment enhances autophagy in RPE cells as a protective mechanism triggered under inflammatory conditions. Furthermore, we demonstrated that the PLD pathway modulates the autophagic process in RPE cells. Further experiments are certainly needed to fully elucidate the mechanisms by which classical PLDs modulates autophagy in RPE cells exposed to inflammatory injury. Our findings contribute to the knowledge of the molecular basis of retinal inflammatory and degenerative diseases, such as AMD, DR and endophthalmitis and open possible avenues of therapeutic exploration.

## Author Contributions

VB and PT performed the experiments. VB and MM designed the experiments and wrote the manuscript. MM supervised the study. NG provided equipment and reagents and revised the manuscript.

## Conflict of Interest Statement

The authors declare that the research was conducted in the absence of any commercial or financial relationships that could be construed as a potential conflict of interest.

## Supplementary Material

FIGURE S1 Confocal images showing NFκB (p65) subcellular distribution in D407 cells exposed to LPS (10 μg/ml) or to control condition (vehicle) for 24 h. Cells were stained with TO-PRO-3 to visualize the nuclear structure. Scale bar = 10 μm.

FIGURE S2 Immunofluorescence assays of ARPE-19 cells. LC3B-positive punctate structures were analyzed by wide-field fluorescence microscopy in ARPE-19 cells exposed for 24 h to 25 μg/ml LPS or control condition (vehicle), with or without FBS. Cells were stained with DAPI to visualize the nuclear structure. Scale bar = 10 μm.

## Abbreviations

3-MA: 3-methyladenine
BAF: bafilomycin A_1_
BECN1: beclin1
COX-2: cyclooxygenase-2
DAG: diacylglycerol
ERK: extracellular signal-regulated kinase
HRP: horseradish peroxidase
LC3: microtubule-associated protein 1 light chain 3
LPS: lipopolysaccharide
LPPs: lipid phosphate phosphatases
mTOR: mammalian target of rapamycin
NFκB: nuclear factor kappa B
RAP: rapamycin
SQSTM1/p62: sequestosome 1
PA: phosphatidic acid
PC: phosphatidylcholine
PGs: prostaglandins
PKC: protein kinase C
PKD: protein kinases D
PLD: phospholipase D
POS: photoreceptor outer segments
PR: photoreceptor
PVDF: polyvinylidene fluoride
RasGRP: Ras guanine-releasing protein
RPE: retinal pigment epithelium
WB: western blot.
