# Supplementary figures and images for: Lipopolysaccharide-Induced Autophagy Mediates Retinal Pigment Epithelium Cells Survival. Modulation by the Phospholipase D Pathway
Source: Front Cell Neurosci. 2019 Apr 24;13:154. doi: 10.3389/fncel.2019.00154 (PMC6497095; doi:10.3389/fncel.2019.00154)

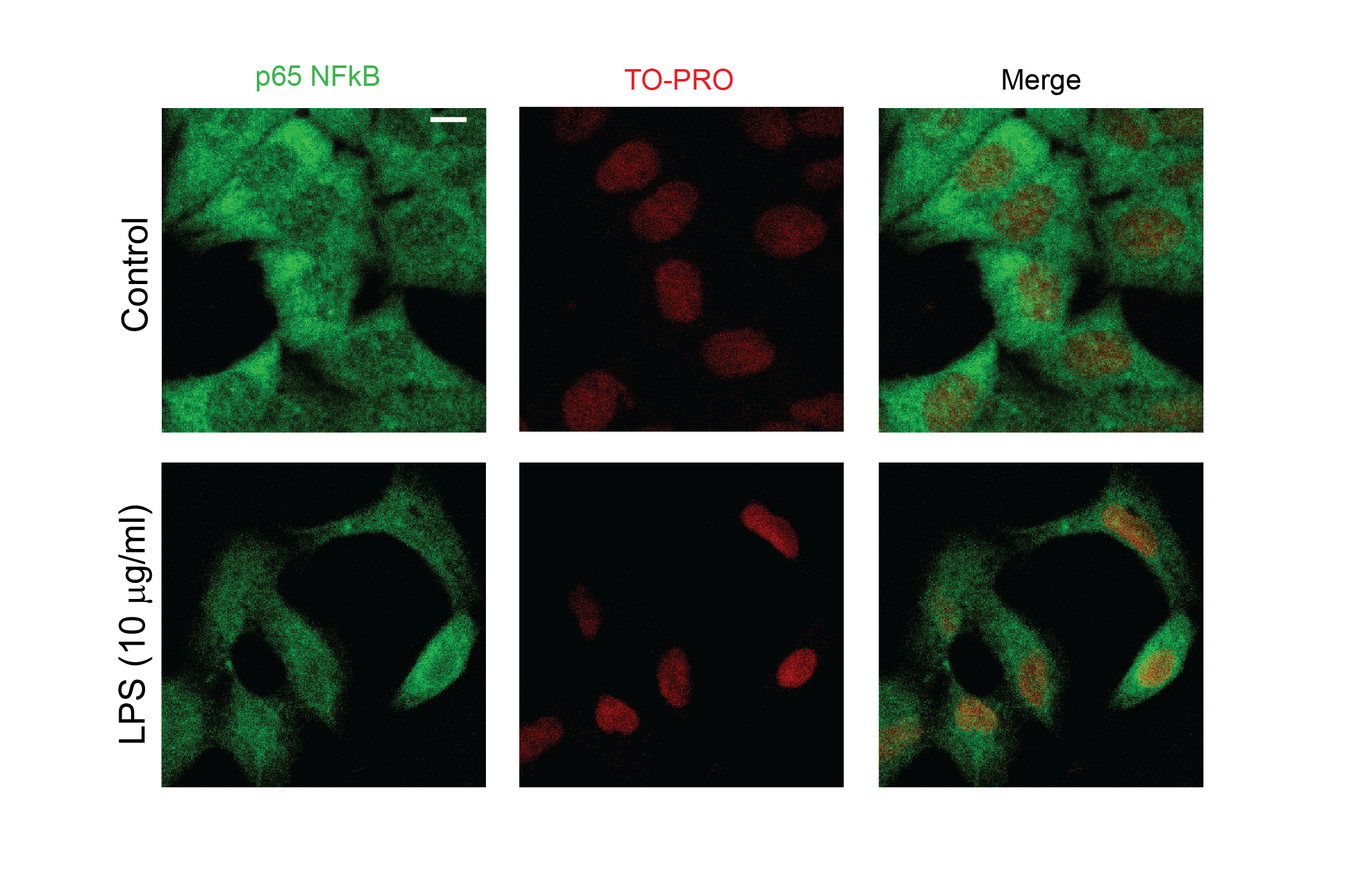

Supplement: FIGURE S1 — Confocal images showing NFκB (p65) subcellular distribution in D407 cells exposed to LPS (10 μg/ml) or to control condition (vehicle) for 24 h. Cells were stained with TO-PRO-3 to visualize the nuclear structure. Scale bar = 10 μm. [file Image_1.TIF]

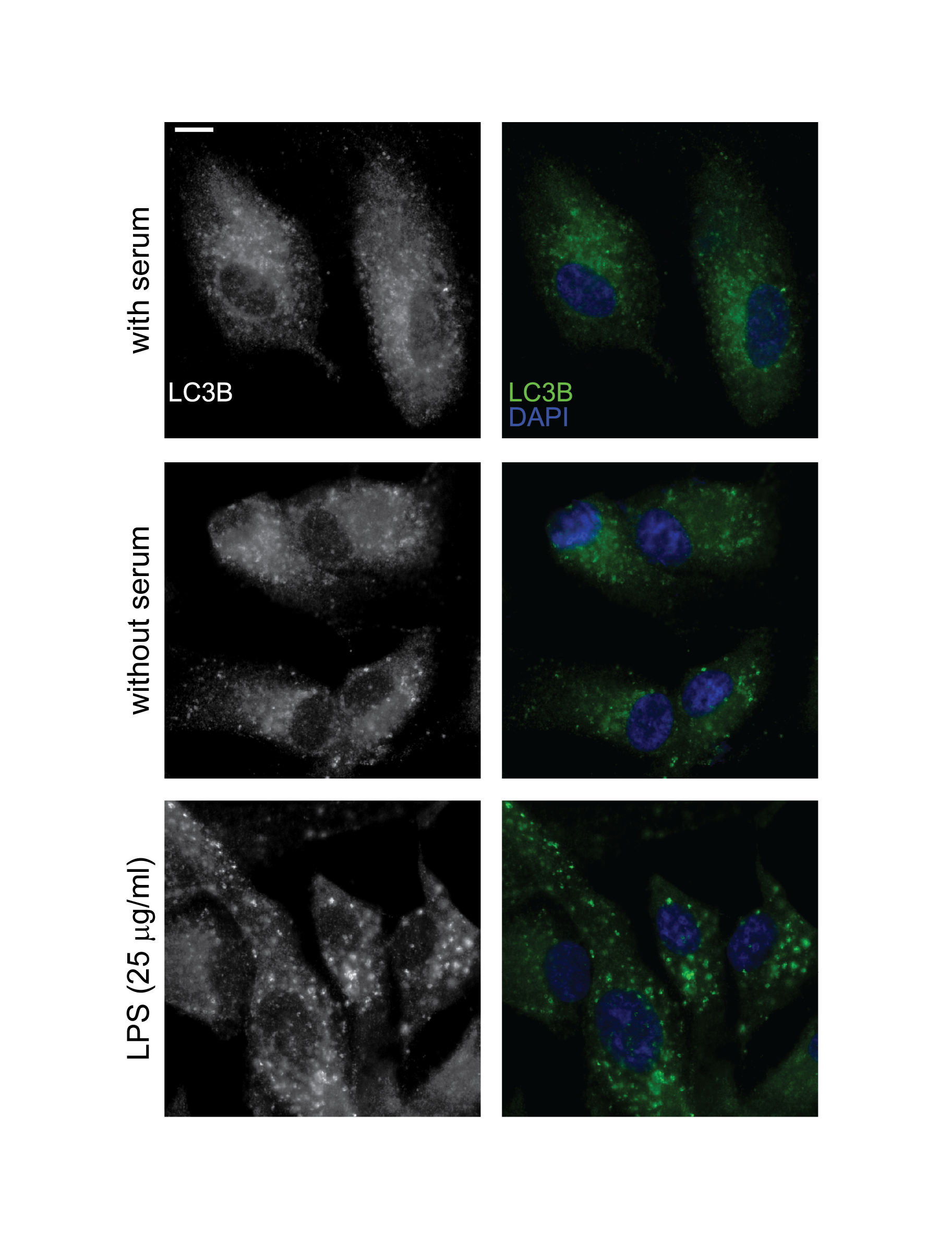

Supplement: FIGURE S2 — Immunofluorescence assays of ARPE-19 cells. LC3B-positive punctate structures were analyzed by wide-field fluorescence microscopy in ARPE-19 cells exposed for 24 h to 25 μg/ml LPS or control condition (vehicle), with or without FBS. Cells were stained with DAPI to visualize the nuclear structure. Scale bar = 10 μm. [file Image_2.TIF]
